# On the Wind of a Cannon-Ball

**Published:** 1817-10

**Authors:** 

**Affiliations:** of the Royal Marines.


THE LONDON
### Medical and Physical Journal.

4 OF VOL. XXXVIII.] OCTOBER, 1817. [NO. 224.

"For many fortunate discoveries in medicine, and for the detection of nume-
"rous errors, the world is indebted to the rapid circulation of Monthly
"Journals; and there never existed any work to which the Faculty in
"EUROPE and AMERICA were under deeper obligations than to the
"Medical and Physical Journal of London, now forming a long, but an
"invaluable, series."—RUSH.

*For the London Medical and Physical Journal.*

### *On the Wind of a Cannon-ball;* by Captain BAGNOLD, of the Royal Marines.

OBSERVING in the New Monthly Magazine, I think for June last, an account of the death of a naval officer by "the wind of a shot," I beg leave to offer the following observations and facts on the subject.

When in battle a man falls dead without any apparent injury, the seamen universally attribute it to this active but invisible agency; and as, from the prejudices of that class of men, dissection cannot often be had recourse to, the real cause remains undiscovered, and the killed are committed to the deep without much philosophical reasoning on the subject. But, if this cause could possibly produce so violent an effect, how is it in the course of war that we meet with frequent instances of men having their arms carried from their sides by cannon-shots from the wrist up to the very shoulder-joint, and yet escape with life? At the taking of the Island of Bourbon, an officer lost his epaulet in this way; a friend of mine part of his hat; a soldier in Sir Thomas Picton's division, at Waterloo, his knapsack; and a still stronger case is that of Colonel J. W——, who, about thirty-six years ago, at St. Lucia, lost his eye-sight from the close passage of a ball.—Surely, in all these cases, the projectile must have passed sufficiently close to the nobler parts to have done all the injury of which it was capable. I shall now abandon inference, and submit the four following cases; the first three occurred under my own eye; and the fourth is from the narration of a scientific friend, a former surgeon of his Majesty's ship Imperieuse, a gentleman whose acquirements and accurate observation entitle him to every attention. From these cases I infer, first, that death may be produced by a wound, which (in the confusion always attendant on such scenes) may escape the eye of a careful examiner; secondly, that parts of the human body, and other substances directly exposed to the lateral action of a shot, frequently suffer less injury than those immediately under them; and that injury sufficient to destroy life may be inflicted while the integuments remain sound.

Case I.—In the year 1800, being employed in the boats of his Majesty's ship Retribution, cutting out a brig from Trinidad in the Island of Cuba, within pistol-shot of the batteries, a short time after we had gained possession of her, one of my men, John Freize, was found in the cabin apparently dead, and expired soon after. On the return of the boats, the body was taken on board the ship, and, being carefully examined, no external injury could be found. The surgeon, however, determining to satisfy his mind on the point, had the head shaved, when a wound was discovered scarcely one-tenth of an inch in diameter; on dissection, the point end of a small nail was found lodged in the brain, with a considerable quantity of extravasated blood; the nail must have been driven there from the vessel's side by one of several shots that passed through her.

Case II.—On the 18th of July 1805, his Majesty's ship Arab, being in action with a division of the enemy's flotilla, within five hundred yards of Cape Grisney, near Boulogne, a thirteen-inch shell was thrown on-board. The velocity of its descent was mitigated by the resistance it met with in passing through the main-top, the treple-trees, jeer-block, and, finally, through the booms down to the main deck, where it made a considerable concave indentation on a large oak-beam. A seaman, named Armstrong, was squatting down at the moment in the act of pointing a gun, the shell fell close behind him, and the man dropped motionless. He complained of pain in his back and inability to move or sit upright, yet there was not the slightest injury or discolouration of the integuments visible. On examination, *post mortem*, it was evident the shell in its descent must have grazed down the lower part of the back, as the spinal processes in that organ were destroyed; the enemy having thrown the shell so short a distance with a long fuse, it was thrown overboard by the heroic exertions of the late Mr. Edward Wogan Mansel, assisted by some of the crew, for which gallant exertion they were liberally rewarded by the Patriotic Fund.

Case III.—In the same action, a seaman, named Woodcock, received a severe injury, as was supposed, from a shot grazing across his stomach. Symptoms of severe inflammation of that organ supervened, and the next day the integuments assumed a bruised coffee-coloured appearance; his cloaths were not in the least injured.

Case IV.—A seaman in his Majesty's ship Imperieuse, commanded by Lord Cochrane, whilst firing at a battery on the coast of France, fell from the effect of a shot passing across the abdomen. The blood immediately showed itself through his canvass trowsers, which remained uninjured; on removing this part of his dress, the muscular substance of the parietes were found nearly destroyed.

If the foregoing facts should throw any additional light on this hitherto controverted subject, I shall feel much pleasure.

*August* 1817.

---

*For the London Medical and Physical Journal.*

